# Deep Learning for Stock Market Prediction

**DOI:** 10.3390/e22080840

**Published:** 2020-07-30

**Authors:** M. Nabipour, P. Nayyeri, H. Jabani, A. Mosavi, E. Salwana, Shahab S.

**Affiliations:** 1 Faculty of Mechanical Engineering, Tarbiat Modares University, Tehran 14115-143, Iran; Mojtaba.nabipour@modares.ac.ir; 2 School of Mechanical Engineering, College of Engineering, University of Tehran, Tehran 1439956153, Iran; pnnayyeri@ut.ac.ir; 3 Department of Economics, Payame Noor University, West Tehran Branch, Tehran 1455643183, Iran; h.jabani@gmail.com; 4 Faculty of Civil Engineering, Technische Universität Dresden, 01069 Dresden, Germany; 5 Department of Informatics, J. Selye University, 94501 Komarno, Slovakia; 6 Institute of IR4.0, Universiti Kebangsaan Malaysia, Bangi 43600, Malaysia; elysalwana@ukm.edu.my; 7 Institute of Research and Development, Duy Tan University, Da Nang 550000, Vietnam

**Keywords:** stock market prediction, machine learning, regression analysis, tree-based methods, deep learning, long short-term memory, LSTM, business intelligence, finance, stock market, financial forecast, information economics, economics, information science

## Abstract

The prediction of stock groups values has always been attractive and challenging for shareholders due to its inherent dynamics, non-linearity, and complex nature. This paper concentrates on the future prediction of stock market groups. Four groups named diversified financials, petroleum, non-metallic minerals, and basic metals from Tehran stock exchange were chosen for experimental evaluations. Data were collected for the groups based on 10 years of historical records. The value predictions are created for 1, 2, 5, 10, 15, 20, and 30 days in advance. Various machine learning algorithms were utilized for prediction of future values of stock market groups. We employed decision tree, bagging, random forest, adaptive boosting (Adaboost), gradient boosting, and eXtreme gradient boosting (XGBoost), and artificial neural networks (ANN), recurrent neural network (RNN) and long short-term memory (LSTM). Ten technical indicators were selected as the inputs into each of the prediction models. Finally, the results of the predictions were presented for each technique based on four metrics. Among all algorithms used in this paper, LSTM shows more accurate results with the highest model fitting ability. In addition, for tree-based models, there is often an intense competition between Adaboost, Gradient Boosting, and XGBoost.

## 1. Introduction

The prediction process of stock values is always a challenging problem [1] because of its long-term unpredictable nature. The dated market hypothesis believes that it is impossible to predict stock values and that stocks behave randomly, but recent technical analyses show that most stocks values are reflected in previous records; therefore the movement trends are vital to predict values effectively [2]. Moreover, stock market groups and movements are affected by several economic factors such as political events, general economic conditions, commodity price index, investors’ expectations, movements of other stock markets, the psychology of investors, etc. [3]. The value of stock groups is computed with high market capitalization. There are different technical parameters to obtain statistical data from the value of stock prices [4]. Generally, stock indices are gained from prices of stocks with high market investment and they often give an estimation of the economic status in each country. For example, findings prove that economic growth in countries is positively impacted by the stock market capitalization [5]. The nature of the stock values movement is ambiguous and makes investments risky for investors. In addition, it is usually difficult to detect the market status for governments. Indeed, the stock values are generally dynamic, non-parametric, and non-linear; therefore, they often cause the weak performance of the statistical models and disability to predict the accurate values and movements [6,7].

Popular theories suggest that stock markets are essentially a random walk, especially when it comes to the Iranian stock market, which comes with some rules of the close price of the previous day. Most conventional time series prediction methods are based on stationary trends; hence the prediction of stock prices deal with inherent difficulty. In addition, predicting stock prices is a challenging problem in itself because of the number of variables that are involved. In short term, the market behaves similar to a voting machine, but in the longer term, it acts similar to a weighing machine and hence there is scope for predicting the market movements for a longer timeframe [8]. Machine learning (ML) is the most powerful tool which includes different algorithms to effectively develop their performance on a certain case study. It is a common belief that ML has a significant ability to identify valid information and detecting patterns from the dataset [9]. In contrast with the traditional methods in the ML area, the ensemble models are a machine learning-based way in which some common algorithms are used to work out a particular problem, and have been confirmed to outperform each of the methods when predicting time series [10,11,12]. For prediction problems in the machine learning area, boosting and bagging are effective and popular algorithms among ensemble ways. There is recent progress of tree-based models with introducing gradient boosting and XGBoost algorithms, which have been significantly employed by top data scientists in competitions. Indeed, a modern trend in ML, which is named deep learning (DL), can deem a deep nonlinear topology in its specific structure, and has an excellent ability from the financial time series to extract relevant information [13]. Contrary to a simple artificial neural network, recurrent neural networks (RNNs) have achieved considerable success in the financial area on account of their great performance [14,15]. It is clear that the prediction process of the stock market is not only related to the current information, but the earlier data have a vital role, so the training will be insufficient if only the data are used at the latest time. RNN can employ the network to sustain the memory of recent events and build connections between each unit of a network, so, it is completely proper for the economic predictions [16,17]. Long short-term memory (LSTM) is an improved subset of the RNN method that used in the deep learning area. LSTM has three different gates to remove the problems in RNN cells and can also process single data points or whole sequences of data.

In academic fields, many studies have been conducted on market prediction ways. Long et al. [18] examined a deep neural network model with public market data and the transaction records to evaluate stock price movement. The experimental results showed that bidirectional LSTM could predict the stock price for financial decisions, and the method acquired the best performance compared to other prediction models. Pang et al. [19] tried to improve an innovative neural network method to get better stock market predictions. They proposed LSTM with an embedded layer and LSTM with an automatic encoder to evaluate the stock market movement. The results showed that the deep LSTM with embedded layer outperformed and the model’s accuracies for the Shanghai composite index are 57.2% and 56.9%, respectively. Kelotra and Pandey [20] used the deep convolutional LSTM model as a predictor to effectively examine stock market movements. The model was trained with a Rider-based monarch butterfly optimization algorithm and they achieved a minimal MSE and RMSE of 7.2487 and 2.6923. Bouktif et al. [21] investigated the predictability of the stock market trend direction with an improved way of sentiments analysis. As the final result, the proposed method outperformed adequately and predicted stock trends with higher accuracy of 60% in comparison with other sentiment-based stock market prediction methods involving deep learning. Zhong and Enke [22] proposed a big comprehensive data of the SPDR S&P 500 ETF to evaluate return direction with 60 economic and financial features. Deep neural networks and artificial neural networks (ANN) were employed via principal component analysis (PCA) to predict the daily future of stock market index returns. The results showed that deep neural networks were superior as classifiers based on PCA-represented data compared to others. Das et al. [23] implemented the feature optimization through considering the social and biochemical aspects of the firefly method. In their approach, they involved the objective value selection in the evolutionary context. The results indicated that firefly, with an evolutionary framework applied to the Online Sequential Extreme Learning Machine (OSELM) prediction method, was the best model among other experimented ones. Hoseinzade and Haratizadeh [24] proposed a Convolutional Neural Networks (CNNs) framework, which can be applied to various data collections (involving different markets) to explore features for predicting the future movement of the markets. From the results, remarkable improvement in prediction’s performance in comparison with other recent baseline methods was achieved. Krishna Kumar and Haider [25] compared the performance of single classifiers with a multi-level classifier, which was a hybrid of machine learning techniques (such as decision tree, support vector machine, and logistic regression classifier). The experimental results revealed that the multi-level classifiers outperformed the other works and led to a more accurate model with the best predictive ability, roughly 10 to 12% growth inaccuracy. Chung and Shin [26] applied one of the deep learning methods (CNNs) for predicting the stock market movement. In addition, the Genetic algorithm (GA) was employed to optimize the parameters of the CNN method systematically, and results showed that the GA-CNN outperformed the comparative models as the hybrid method of GA and CNN. Sim et al. [27] proposed CNN to predict stock prices as a new learning method. The study aimed to solve two problems, using CNNs and optimizing them for stock market data. Wen et al. [28] applied the CNN algorithm on noisy temporal series by frequent patterns as a new method. The results proved that the method was adequately effective and outperformed traditional signal process methods with a 4 to 7% accuracy improvement.

Rekha et al. [29] employed CNN and RNN to make a comparison between two algorithms’ results and actual results via stock market data. Lee et al. [30] used CNNs to predict the global stock market and then trained and tested their model with data from other countries. The results demonstrated that the model could be trained on the relatively large data and tested on the small markets where there was not enough amount of data. Liu et al. [31] investigated a numerical-based attention method with dual sources stock market data to find the complementarity between numerical data and news in the prediction of stock prices. As a result, the method filtered noises effectively and outperformed prior models in dual sources stock prediction. Baek and Kim [32] proposed an approach for stock market index forecasting, which included a prediction LSTM module and an overfitting prevention LSTM module. The results confirmed that the proposed model had an excellent forecasting accuracy compared to a model without an overfitting prevention LSTM module. Chung and Shin [33] employed a hybrid approach of LSTM and GA to improve a novel stock market prediction model. The final results showed that the hybrid model of the LSTM network and GA was superior in comparison with the benchmark model. Chen et al. [34] used three neural networks, the radial basis function neural network, the extreme learning machine, and three traditional artificial neural networks, to evaluate their performance on high-frequency data of the stock market. Their results indicated that deep learning methods got transaction data from the nonlinear features and could predict the future of the market powerfully. Zhou et al. [35] applied LSTM and CNN on high-frequency data from the stock market with the approach of rolling partition training and testing set to evaluate the update cycle effect on the performance of models. Based on extensive experimental results, Models could effectively reduce errors and increase prediction accuracy. Chong et al. [36] tried to examine the performance of deep learning algorithms for stock market prediction with three unsupervised feature extraction ways, PCA, restricted Boltzmann machine and auto encoder. Final results with significant improvement suggested that additional information could be extracted by deep neural networks from the autoregressive model.

Long et al. [37] suggested an innovative end-to-end model named multi-filters neural network (MFNN) specifically for price prediction task and feature extraction on financial time series data. Their results indicated that the network outperformed common machine learning methods, statistical models, and convolutional, recurrent, and LSTM networks in terms of accuracy, stability and profitability. Moews et al. [38] proposed employing deep neural networks that use step-wise linear regressions in the preparatory feature engineering with exponential smoothing for this task, with regression slopes as movement strength indicators for a specified time interval. The final results showed the feasibility of the suggested method, with advanced accuracies and accounting for the statistical importance of the results for additional validation, as well as prominent implications for modern economics. Garcia et al. [39] examined the effect of financial indicators on the German DAX-30 stock index by employing a hybrid fuzzy neural network to forecast the one-day ahead direction of the index with various methods. Their experimental works demonstrated that the fall in the dimension through the factorial analysis produces less risky and profitable strategies. Cervelló-Royo and Guijarro [40] compared the performance of four machine learning models to validate the predicting capability of technical indicators in the technological NASDAQ index. The results showed that the random forest outperformed the other models deemed in their study, being able to predict the 10-days ahead market movement, with a normal accuracy of 80%. Konstantinos et al. [41] suggested an ensemble prediction combination method as an alternative approach to forecast time series. The ensemble learning technique combined various learning models. Their results indicated the effectiveness proposed ensemble learning way, and the comparative analysis showed adequate evidence that the method could be used successfully to conduct prediction based on multivariate time series problems.

Overall, all researchers believe that stock price prediction and modeling have been challenging problems for study and speculators due to noisy and non-stationary characteristics of data. There is a minor difference between papers for choosing the most effective indicators for modeling and predicting the future of stock markets. Feature selection can be an important part of studies to achieve better accuracy; however, all studies indicate that uncertainty is an inherent part of these forecasting tasks because of fundamental variables. Employing new machine learning and deep learning methods such as recent ensemble learning models, CNNs and RNNs with high prediction ability is a significant advantage of recent studies that show the forecasting potential of these methods in comparison with traditional and common approaches such as statistical analyses.

Iran’s stock market has been highly popular recently because of the arising growth of Tehran Stock Exchange Dividend and Price Index-TEDPIX in the last decades, and one of the reasons is that most of the state-owned firms are being privatized under the general policies of article 44 in the Iranian constitution, and people are allowed to buy the shares of newly privatized firms under the specific circumstances. This market has some specific attributes in comparison with other country’s stock markets, one of them being dealing a price limitation of ±5% of the opening price of the day for every index. This issue hinders the abnormal market fluctuation and scatters market shocks, political issues, etc. over a specific time and could make the market smoother and more predictable. Trading takes place through licensed registered private brokers of exchange organization and the opening price of the next day is through the defined base-volume of the companies and transaction volume as well. However, the deficiency of valuable papers on this market to predict future values with machine learning models is clear.

This study concentrates on the process of future value prediction for stock market groups, which are crucial for investors. Despite significant development in Iran’s stock market in recent years, there has been not enough research on the stock price predictions and movements using novel machine learning methods. This paper aims to compare the performance of some regressors which applied on fluctuating data to evaluate predictor models, and the predictions are evaluated for 1, 2, 5, 10, 15, 20, and 30 days in advance. In addition, with tuning parameters, we try to reduce errors and increase the accuracy of models.

Ensemble learning models are broadly employed nowadays for its predictive performance progress. These methods combine multiple forecasts from one or multiple methods to improve the accuracy of simple prediction and to prevent possible overfitting problems. In addition, ANNs are universal approximators and flexible computing frameworks which can be used to an extensive range of time series predicting problems with a great degree of accuracy. Therefore, by considering the literature review, this research work examines the predictability of a set of cutting-edge machine learning methods, which involves tree-based models and deep learning methods. Employing the whole of tree-based methods, RNN, and LSTM techniques for regression problems and comparing their performance in Tehran stock exchange is a recent research activity presented in this study. This paper includes three different sections. At first, through the methodology section, the evolution of tree-based models with the introduction of each one is presented. Besides, the basic structure of neural networks and recurrent ones are described briefly. In the research data section, 10 technical indicators are shown in detail with selected methods parameters. At the final step, after introducing three regression metrics, machine learning results are reported for each group, and the model’s behavior is compared.

## 2. Materials and Methods

### 2.1. Tree-Based Models

**Figure 1:**
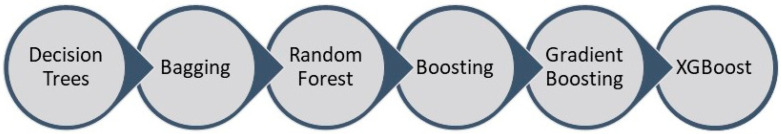
The evolution of tree-based methods.

Since the set of splitting rules employed to differently divide the predictor space can be summarized in a tree, these types of models are known as decision-tree methods. Figure 1, adapted from [42,43] shows the evolution of tree-based algorithms over several years and the following sections introduce them.

**Figure 2:**
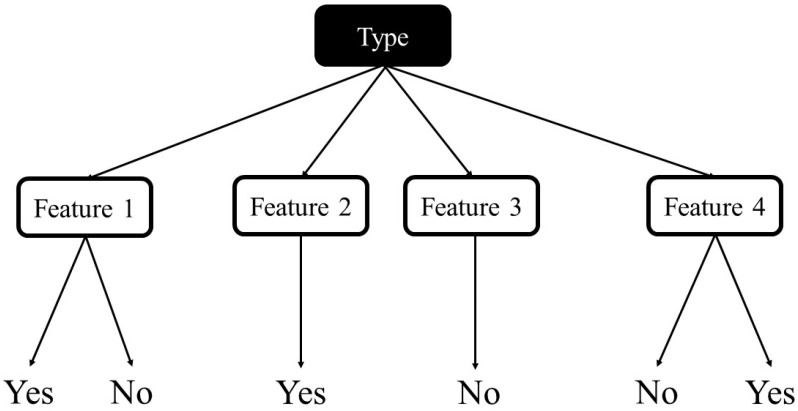
Schematic illustration of decision tree

Decision Trees are a popular supervised learning technique used for classification and regression jobs. The purpose is to make a model that predicts a target value by learning easy decision rules formed from the data features. There are some advantages of using this method, such as it being easy to understand and interpret or able to work out problems with multi-outputs; on the contrary, creating over-complex trees that result in overfitting is a fairly common disadvantage. A schematic illustration of the Decision tree is shown in Figure 2, adapted from [43].

A Bagging model (as a regressor model) is an ensemble estimator that fits each basic regressor on random subsets of the dataset and next accumulate their single predictions, either by voting or by averaging, to make the final prediction. This method is a meta-estimator and can commonly be employed as an approach to decrease the variance of an estimator such as a decision tree by using randomization into its construction procedure and then creating an ensemble out of it. In this method, samples are drawn with replacement and predictions and obtained through a majority voting mechanism.

**Figure 3:**
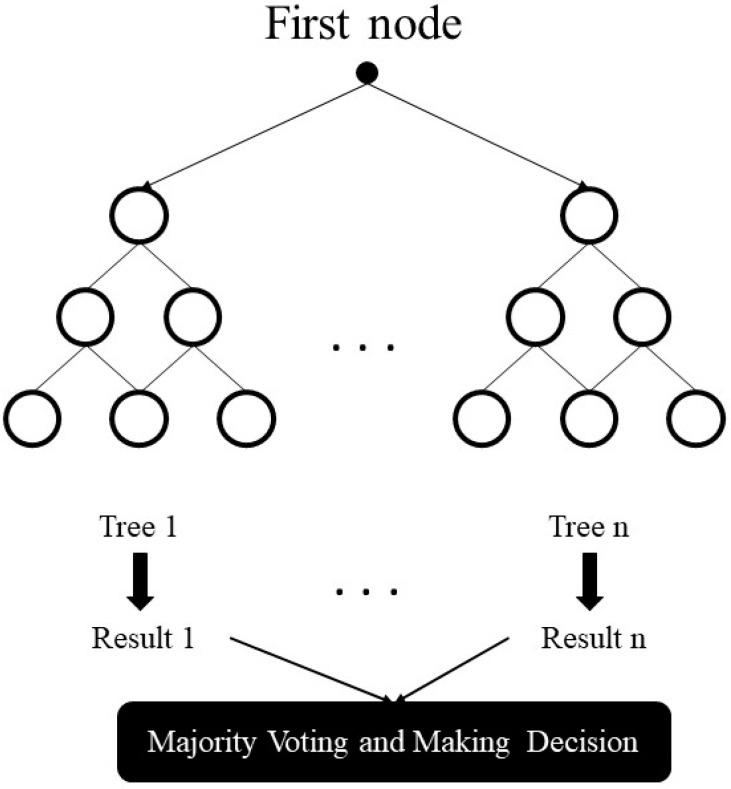
Schematic illustration of the random forest.

The random forest model is created by a great number of decision trees. This method simply averages the prediction result of trees, which is called a forest. In addition, this model has three random concepts; randomly choosing training data when making trees, selecting some subsets of features when splitting nodes, and considering only a subset of all features for splitting each node in each simple decision tree. During training data in a random forest, each tree learns from a random sample of the data points. A schematic illustration of the random forest, adapted from [43], is indicated in Figure 3.

The boosting method refers to a group of algorithms that converts weak learners to a powerful learner. The method is an ensemble for developing the model predictions of any learning algorithm. The concept of boosting is to sequentially train weak learners to correct their past performance. AdaBoost is a meta-estimator that starts by fitting a model on the main dataset and then fits additional copies of the model on a similar dataset. During the process, the samples’ weights are adapted based on the current prediction error, so subsequent models concentrate more on difficult items. Gradient Boosting method is similar to AdaBoost when it sequentially adds predictors to an ensemble model, each of them correcting its past performance. In contrast with AdaBoost, Gradient Boosting fits a new predictor of the residual errors (made by the prior predictor) using gradient descent to find the failure in the predictions of the previous learner. Overall, the final model is capable of employing the base model to decreases errors over time.

The XGBoost is an ensemble tree method (similar to Gradient Boosting) and the method applies the principle of boosting for weak learners. However, XGBoost was introduced for better speed and performance. In-built cross-validation ability, efficient handling of missing data, regularization for avoiding overfitting, catch awareness, tree pruning, and parallelized tree building are common advantages of the XGBoost algorithm.

### 2.2. Artificial Neural Networks

**Figure 4:**
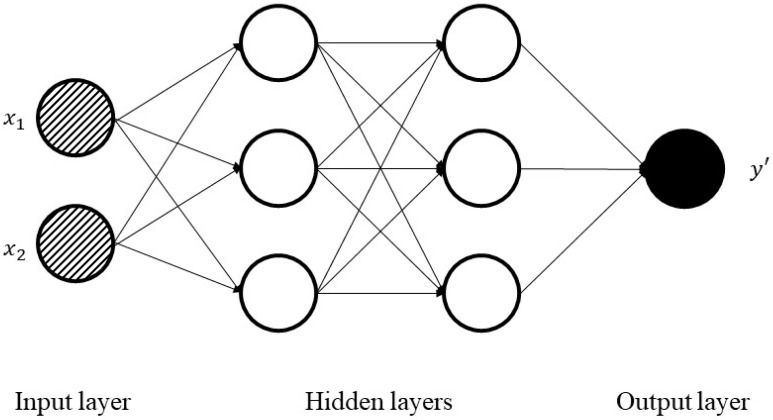
Schematic illustration of artificial neural networks

ANNs are single or multi-layer neural nets that are fully connected. Figure 4 shows a sample of ANN with an input and output layer and also two hidden layers, adapted from [43]. In a layer, each node is connected to every other node in the next layer. With an increase in the number of hidden layers, it is possible to make the network deeper.

**Figure 5:**
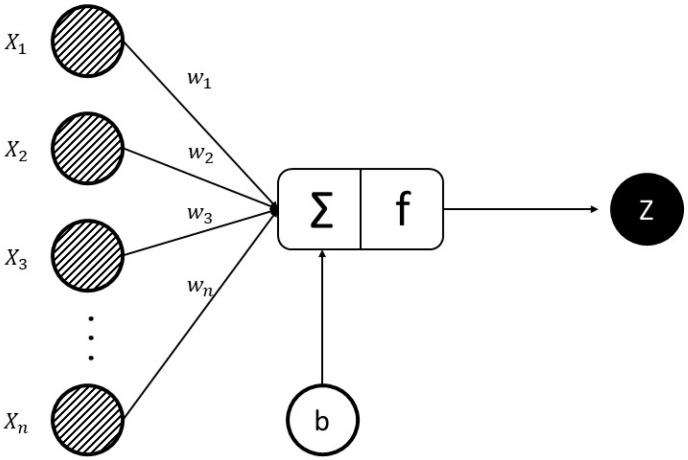
An illustration of relationship between inputs and output for ANN.

Figure 5 is shown for each of the hidden or output nodes, while a node takes the weighted sum of the inputs, added to a bias value, and passes it through an activation function (usually a non-linear function). The result is the output of the node that becomes another node input for the next layer. The procedure moves from the input to the output, and the final output is determined by doing this process for all nodes. The learning process of weights and biases associated with all nodes for training the neural network.

Equation (1) shows the relationship between nodes, weights, and biases [44]. The weighted sum of inputs for a layer passed through a non-linear activation function to another node in the next layer. It can be interpreted as a vector, where X_1_, X_2_, …, and Xn are inputs, w_1_, w_2_, …, and w_n_ are weights respectively, n is the number of the input for the final node, f is activation function and z is the output.

(1) $$Z=f\;(x.w+b)=f\;(\sum_{i=1}^{n}x_{i}^{T}w_{i}+b).$$

By calculating weights/biases, the training process is completed by some rules: initialize the weights/biases for all the nodes randomly, performing a forward pass by the current weights/biases and calculating each node output, comparing the final output with the actual target, and modifying the weights/biases consequently by gradient descent with the backward pass, generally known as backpropagation algorithm.

RNN is a very prominent version of neural networks extensively used in various processes. In a common neural network, the input is processed through several layers, and output is made. It is assumed that two consecutive inputs are independent of each other. However, the situation is not correct in all processes. For example, for the prediction of the stock market at a certain time, it is crucial to consider the previous observations.

**Figure 6:**
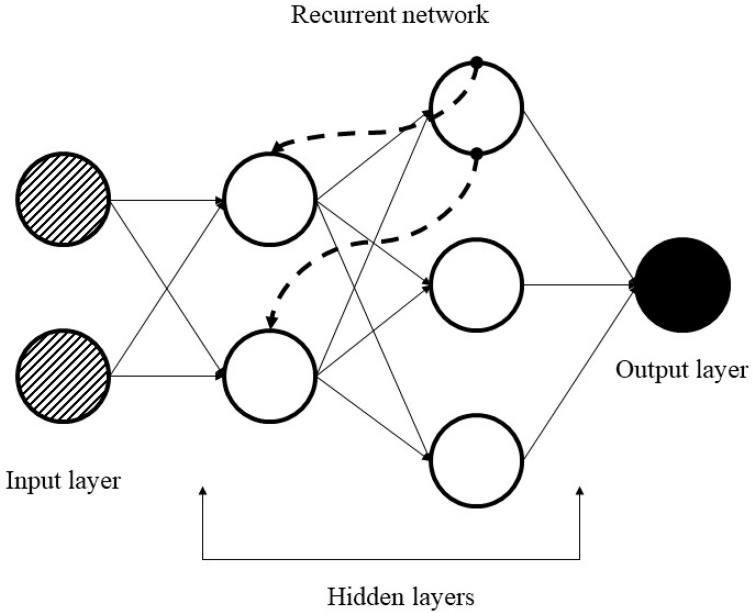
An illustration of recurrent network.

Simple RNN has multiple neurons to create a network. Each neuron has a time-varying activation function and each connection between nodes has a real-valued weight that can be modified at each step. According to general architecture, the output of the node (at time t − 1) will be passed to the input (at time t) and add the data of itself (at time t) to make the output (at time t); recurrently exploiting the neuron node to flow multiple node elements to create RNN. Figure 6, adapted from [43] shows a simple architecture of RNN. Furthermore, Equations (2) and (3) indicate the recursive formulas of RNN [45].

h_t_ = tanh (W_t_h_t−1_ + W_x_x_t_), (2)

y_t_ = W_y_h_t,_ (3)

where y_t_, h_t_, x_t_, and W_h_ are output vector, hidden layer vector, input vector, and weighting matrix respectively

LSTM is a specific kind of RNN with a wide range of applications similar to time series analysis, document classification, speech, and voice recognition. In contrast with feedforward ANNs, the predictions made by RNNs are dependent on previous estimations. In real, RNNs are not employed extensively because they have a few deficiencies which cause impractical evaluations. The difference between LSTM and RNN is that every neuron in LSTM is a memory cell. The LSTM links the prior information to the current neuron. Every neuron has three gates (input gate, forget gate, and output gate). By the internal gate, the LSTM is able to solve the long-term dependence problem of the data. LSTM architecture includes forget gate, input gate, and output gate. The forget gate controls discarding information from the cell, and Equations (4) and (5) show its related formulas where h_t−1_ is output at the prior time (t − 1), and x_t_ is input at the current time (t) into Sigmoid function (S(t)). All W and b are the weight matrices and bias vectors that require to be learned during the training process. f_t_ defines how much information will be remembered or forgotten. The input gate defines which new information remember in cell state by Equations (5)–(7). the value of i_t_ is generated to determine how much new information cell state need to be remembered. A tanh function gains an election message to be added to the cell state by inputting the output (h_t−1_) at the prior time (t − 1) and adding the current time t input information (x_t_). C_t_ gets the updated information that must be added to the cell state (Equation (8)). The output gate defines which information will be output in cell state. The value of o_t_ is between 0 and 1; which is employed to indicate how many cells state information that need to output (Equation (9)). The result of h_t_ is the LSTM block’s output information at time t (Equation (10)) [45].

ft = σ (W_f_ · [h_t−1_, x_t_] + b_f_) (4)

(5) $$S(t)=\frac{1}{1+e^{-t}}$$

i_t_ = σ(W_i_ · [h_t−1_, x_t_] + b_i_) (6)

Ĉ_t_ = tanh(W_c_ · [h_t−1,_ x_t_] + b_c_) (7)

C_t_ = f_t_ × C_t−1_ + i_t_ × Ĉ_t_ (8)

o_t_ = σ(W_o_ · [h_t−1_, x_t_] + b_o_) (9)

h_t_ = o_t_ × tanh(C_t_) (10)

## 3. Research Data

This study aims to make a short run prediction for the emerging Iranian stock market and employ data from November 2009 to November 2019 (10 years) of four stock market groups, Diversified Financials, Petroleum, Non-metallic minerals, and Basic metals, which are completely generous. From the opening, close, low high, and prices of the groups, 10 technical indicators are calculated. The data for this study is supplied from the online repository of the Tehran Securities Exchange Technology Management Co (TSETMC) [46]. Before using information for the training process, it is vital to take a preprocessing step. We employ data cleaning, which is the process of detecting and correcting inaccurate records from a dataset and refers to identifying inaccurate or irrelevant parts of the data and then replacing, modifying, or deleting the dirty data. The interquartile range (IQR score) is a measure of statistical dispersion and is robust against outliers, and this method is used to detect outliers and modify the dataset. Indeed, as an important point, to prevent the effect of the larger value of an indicator on the smaller ones, the values of 10 technical indicators for all groups are normalized independently. Data normalization refers to rescaling actual numeric features into a 0 to 1 range and is employed in machine learning to create a training model less sensitive to the scale of variables. Table 1 indicates all the technical indicators, which are employed as input values based on domain experts and previous studies [47,48,49]; the input values for calculating indicators in the table are opening, high, low and closing prices in each trading day; “t” means current time, and “t + 1” and “t − 1” mean one day ahead and one day before, respectively. Table 2 shows the summary statistics of indicators for the groups.

SMA is calculated by the average of prices in a selected range, and this indicator can help to determine if a price will continue its trend. WMA gives us a weighted average of the last n values, where the weighting falls with each prior price. MOM calculates the speed of the rise or falls in stock prices and it is a very useful indicator of weakness or strength in evaluating prices. STCK is a momentum indicator over a particular period of time to compare a certain closing price of a stock to its price range. The oscillator sensitivity to market trends can be reduced by modifying that time period or by a moving average of results. STCD measures the relative position of the closing prices in comparison with the amplitude of price oscillations in a certain period. This indicator is based on the assumption that as prices increase, the closing price tends towards the values which belong to the upper part of the area of price movements in the preceding period and when prices decrease, the opposite is correct. LWR is a type of momentum indicator which evaluates oversold and overbought levels. Sometimes LWR is used to find exit and entry times in the stock market. MACD is another type of momentum indicator which indicates the relationship between two moving averages of a share’s price. Traders can usually use it to buy the stock when the MACD crosses above its signal line and sell the shares when the MACD crosses below the signal line. ADO is usually used to find out the flow of money into or out of stock. ADO line is normally employed by traders seeking to determine buying or selling time of stock or verify the strength of a trend. RSI is a momentum indicator that evaluates the magnitude of recent value changes to assess oversold or overbought conditions for stock prices. RSI is showed as an oscillator (a line graph which moves between two extremes) and moves between 0 to 100. CCI is employed as a momentum-based oscillator to determine when a stock price is reaching a condition of being oversold or overbought. CCI also measures the difference between the historical average price and the current price. The indicator determines the time of entry or exit for traders by providing trade signals.

Dataset used for all models—except RNN and LSTM models—are identical. There are 10 features (10 technical indicators) and one target (stock index of the group) for each sample of the dataset. As mentioned, all 10 features are normalized independently before being used to fit models and improve the performance of algorithms. Since the goal is developing models to predict stock group values, datasets are rearranged to incorporate the 10 features of each day to the target value of n-days ahead. In this study, models are evaluated by training them to predict the target value for 1, 2, 5, 10, 15, 20, and 30 days ahead. There are several parameters related to each model, but we tried to choose the most effective ones concerning our experimental works and prior studies. For tree-based models, several trees (ntrees) were the design parameter while other common parameters are set identical between all models. Parameters and their values for each model are listed in Table 3.

The number of trees to perform tree-based models is fairly robust to over-fitting, so a large number typically results in better prediction. The maximum depth of the individual regression estimators limits the number of nodes in the tree. The best value depends on the interaction of the input variables. In machine learning, the learning rate is an important parameter in an optimization method that finds out the step size at each iteration while moving toward a minimum of a loss function. For RNN and LSTM networks, because of their time-series behavior, datasets are arranged to include the features of more than just one day. While for the ANN model, all parameters but epochs are constant; for RNN and LSTM models, the variable parameters are several days included in the training dataset and respective epochs. By increasing the number of days in the training set, the number of epochs is increased to train the models with an adequate number of epochs. Table 4 presents all valid values for the parameters of each model. For example, if five days are included in the training set for ANN, RNN, or LSTM models, the number of epochs is set to 300 to thoroughly train the models.

The activation function of a node in ANNs describes the output of that node given an input or set of inputs. Optimizers are methods employed to change the attributes of ANNs, such as learning rate and weights to reduce the losses. An epoch is a term used in ANNs and shows the number of passes of the entire training dataset the ANN model has completed.

## 4. Evaluation Measures

In this section four metrics used in the study are introduced.

### 4.1. Mean Absolute Percentage Error

Mean Absolute Percentage Error (MAPE) is often employed to assess the performance of the prediction methods. MAPE is also a measure of prediction accuracy for forecasting methods in the machine learning area, it commonly presents accuracy as a percentage. Equation (11) shows its formula [50].

(11) $$\mathrm{MAPE}=\frac{1}{n}\sum_{t=1}^{n}\left|\frac{A_{t}-F_{t}}{A_{t}}\right|\;\times \;100,$$

where *A_t_* is the actual value and *F_t_* is the forecast value. In the formula, the absolute value of the difference between those is divided by *A_t_*. The absolute value is summed for every forecasted value and divided by the number of data. Finally, the percentage error is made by multiplying to 100.

### 4.2. Mean Absolute Error

Mean absolute error (MAE) is a measure of the difference between two values. MAE is an average of the difference between the prediction and the actual values. MAE is a usual measure of prediction error for regression analysis in the machine learning area. The formula is shown in Equation (12) [50,51].

(12) $$\mathrm{MAE}=\frac{1}{n}\sum_{t=1}^{n}\left|A_{t}-F_{t}\right|,$$

where *A_t_* is the true value and *F_t_* is the prediction value. In the formula, the absolute value of the difference between those is divided by *n* (number of samples) and summed for every forecasted value.

### 4.3. Relative Root Mean Square Error

Root Mean Square Error (RMSE) is the standard deviation of the prediction errors in regression work. Prediction errors or residuals show the distance between real values and a prediction model, and how they are spread out around the model. The metric indicates how data is concentrated near the best fitting model. RMSE is the square root of the average of squared differences between predictions and actual observations. Relative Root Mean Square Error (RRMSE) is similar to RMSE and this takes the total squared error and normalizes it by dividing by the total squared error of the predictor model. The formula is shown in Equation (13) [50,51].

(13) $$\mathrm{RRMSE}=\sqrt{\frac{1}{n}\sum_{t=1}^{n}{\left(\;\frac{A_{t}-F_{t}}{A_{t}}\right)}^{2}},$$

where *A_t_* is the observed value, *F_t_* is the prediction value and n is the number of samples.

### 4.4. Mean Squared Error

The Mean Squared Error (MSE) measures the quality of a predictors and its value is always non-negative (values closer to zero are better). The MSE is the second moment of the error (about the origin), and incorporates both the variance of the prediction model (how widely spread the predictions are from one data sample to another) and its bias (how close the average predicted value is from the observation). The formula is shown in Equation (14) [50].

(14) $$\mathrm{MSE}=\frac{1}{n}\sum_{t=1}^{n}{\left(A_{t}-F_{t}\right)}^{2},$$

where *A_t_* is the observed value, *F_t_* is the prediction value and n is the number of samples.

## 5. Results

Six tree-based models namely Decision Tree, Bagging, Random Forest, Adaboost, Gradient Boosting, and XGBoost, and also three neural networks-based algorithms (ANN, RNN, and LSTM) are employed in the prediction of the four stock market groups. For the purpose, prediction experiments for 1, 2, 5, 10, 15, 20, and 30 days in advance of time are conducted. Results for Diversified Financials are depicted in Table 5, Table 6, Table 7, Table 8, Table 9, Table 10 and Table 11 for instance. For better understanding and reduction of the number of result tables, the average performance of algorithms for each group is demonstrated in Table 12, Table 13, Table 14 and Table 15, and also Table 16 shows the average runtime per sample for all models. It is prominent to note that a comprehensive number of experiments are performed for each of the groups and prediction models with various model parameters. The following tables show the best parameters where a minimum prediction error is obtained. Indeed, it is clear from the results that error values generally rise when prediction models are created for a greater number of days ahead. For example, MAPE values of XGBoost are 0.88, 1.14, 1.45, 1.77, 2.03, 2.30, and 2.48 respectively. However, it is possible to observe a less strict ascending trend in some cases (which was seen in previous studies similarly) due to deficiency in the prediction ability of some models in some special cases based on the main dataset.

**Figure 7:**
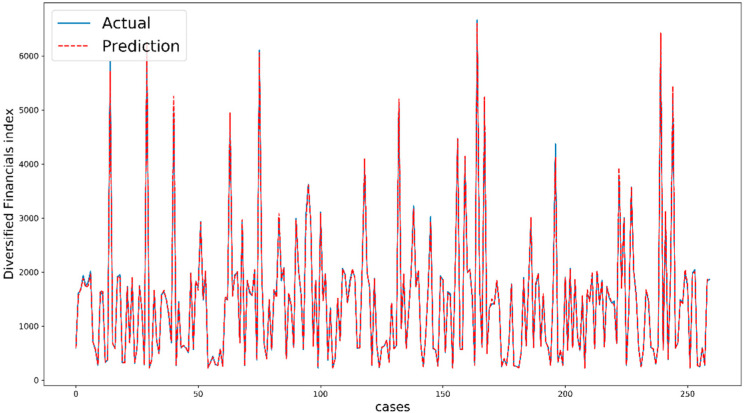
Performance of XGBoost for five days ahead of Diversified Financials.

In this work, we use all of 10 technical indicators as 10 input features and the number of data is 2600. To prevent overfitting, we randomly split our main dataset into two parts, train data and test data, at the first step and then fit our models on the train data. Seventy percent of the main dataset (1820 data) is assigned to train data. Next, the models are used to predict future values and calculate metrics with test data (780 data). In addition, we employ regularization and validation data (20% of train data) to increase our accuracy and tune our hyperparameters during training (the training process for tree-based models and ANNs is different here). Figure 7 shows the performance of XGBoost for five days ahead of Diversified Financials as an example. The comparison between actual values and predicted values indicate the quality of modeling and the prediction task. It is important to note that the cases are not exactly consecutive trading days because we split our dataset randomly by shuffling.

By deeming the literature, our result in this study is one of the most accurate predictions and it can be interpreted by the dataset and the performance of models. It is true that the process of training is totally important, but we believe that the role of the dataset is greater here. The dataset is relatively specific because of some rules in Tehran stock exchange. For example, the value change of each stock is limited to +5% and −5%, or the closing price of a stock is close to the opening price on the next trading day. These rules are learned by machine learning algorithms and then the models are able to significantly predict our dataset from Tehran stock exchange.

Regarding the results of Diversified Financials as an example, Adaboost regressor and LSTM can predict the future prices well with normally 1.59% and 0.60% error; these values become more important when we know that the maximum range of changes is 10% (from −5% to +5%). So, with the specific dataset and powerful models, we still have noticeable errors, which indicate the effect of fundamental parameters. Fundamental analysis is a method of measuring a security’s intrinsic value by examining related economic and financial factors. This method of stock analysis is considered to be in contrast to technical analysis, which forecasts the direction of prices. Noticeably, most non-scientific factors such as policies, increase in tax etc. affect the groups in stock markets; for example, the pharmaceutical industries experience growth with Covid-19 at the present time.

Based on extensive experimental works and reported values the following results are obtained:

Among tree-based models

- Decision Tree always has the lowest rank for predictions because it is not an ensemble method (average of MAPE: 2.07, 2.70, 2.18, 1.41)
- For Diversified Financials and Petroleum, the best average performance belongs to Adaboost regressor (average of MAPE: 1.59 and 2.22)
- For Non-metallic minerals and Basic metals, there is a stiff competition between Adaboost regressor, Gradient Boosting regressor and XGBoost regressor
- Decision Tree has the lowest runtime and then Adaboost regressor is the fastest predictor (0.009 ms and 1.308 ms)
- The runtime of XGBoost is considerably more than other performers (up to 65%)
- Adaboost regressor is the best by considering accuracy, the strength of fitting and runtime all together

Through neural networks

- ANN generally occupies the bottom for forecasting (average of MAPE: 3.86, 5.52, 4.67, 3.17)
- LSTM model outperforms RNN significantly with lower error values (for example in Diversified Financials, MAPE: 0.60 versus 1.85)
- The average runtime of LSTM is noticeably larger than RNN (802.902 ms versus 20.630 ms, roughly four times more)

On the whole

- According to MAPE and RRMSE, the models are able to predict future values for Metals and Diversified Financials better than two other groups
- Deep learning methods (RNN and LSTM) indicate a powerful ability to predict stock market prices because of using a large number of epochs and values related to some days before.
- Based on RRMSE and MSE, the deep learning methods have a high ability to make the best fitting curve with the minimum distribution of residuals around it.
- The average runtime of deep learning models is high compared to others
- LSTM is powerfully the best model for prediction all stock market groups with the lowest error and the best ability to fit, but the problem is the great runtime

In spite of noticeable efforts to find valuable studies on the same stock market, there is not any important paper to report, and this deficiency is one of the novelties of this research. We believe that this paper can be a baseline to compare for future studies.

## 6. Conclusions

For all investors, it is always necessary to predict stock market changes for detecting accurate profits and reducing potential mark risks. This study employed tree-based models (Decision Tree, Bagging, Random Forest, Adaboost, Gradient Boosting, and XGBoost) and neural networks (ANN, RNN, and LSTM) to correctly forecast the values of four stock market groups (Diversified Financials, Petroleum, Non-metallic minerals, and Basic metals) as a regression problem. The predictions were made for 1, 2, 5, 10, 15, 20, and 30 days ahead. As far as our belief and knowledge, this study is the most successful and recent research work that involves ensemble learning methods and deep learning algorithms for predicting stock groups as a popular application. To be more detailed, exponentially smoothed technical indicators and features were used as inputs for prediction. In this prediction problem, the methods were able to significantly advance their performance, and LSTM was the top performer in comparison with other techniques. Overall, as a logical conclusion, both tree-based and deep learning algorithms showed remarkable potential in regression problems to predict the future values of the Tehran stock exchange. Among all models, LSTM was our superior model for predicting all stock market groups with the lowest error and the best ability to fit (by average values of MAPE: 0.60, 1.18, 1.52 and 0.54), but the problem was the great runtime (80.902 ms per sample). As future work, we recommend using the algorithms on other stock markets or examining other hyperparameters effects on the final results.

## Figures and Tables

**Table 1:** Selected technical indicators (n is 10 here).

Simple n-day moving average = $\frac{C_{t}+C_{t-1}+\ldots +C_{t-n+1}}{n}$
Weighted 14-day moving average = $\frac{n*C_{t}+\left(n-1\right)*C_{t-1}+\ldots +C_{t-n+1}}{n+\left(n-1\right)+\ldots +1}$
Momentum = $C_{t}-C_{t-n+1}$
Stochastic K% = $\frac{C_{t}-LL_{t\_\_t-n+1}}{HH_{t\_\_t-n+1}-LL_{t\_\_t-n+1}}$ × 100
Stochastic D% = $\frac{K_{t}+K_{t-1}+\ldots +K_{t-n+1}}{n}$ × 100
Relative strength index (RSI) = 100 − 1001+∑i=1n−1UPt−i∑i=1n−1DWt−i
Signal(n)_t_ = MACD_t_ × $\frac{2}{n+1}$ + Signal(n)_t−1_ × (1 − $\frac{2}{n+1}$)
Larry William’s R% = $\frac{HH_{t\_\_t-n+1}-C_{t}}{HH_{t\_\_t-n+1}-LL_{t\_\_t-n+1}}$ × 100
Accumulation/Distribution (A/D) oscillator: $\frac{H_{t}-C_{t}}{H_{t}-L_{t}}$
CCI (Commodity channel index) = $\frac{M_{t}-SM_{t}}{0.015D_{t}}$
While:
n is number of days
C_t_ is the closing price at time t
L_t_ and H_t_ is the low price and high price at time t, respectively
$LL_{t\_\_t-n+1}$ and $HH_{t\_\_t-n+1}$ is the lowest low and highest high prices in the last n days, respectively
UP_t_ and DW_t_ means upward price change and downward price change at time t, respectively
EMA(K)_t_ = EMA(K)_t−1_ × (1 − $\frac{2}{k+1}$) + C_t_ × $\frac{2}{k+1}$
Moving average convergence divergence (MACD_t_) = EMA(12)_t_ − EMA(26)_t_
M_t_ = $\frac{H_{t}+L_{t}+C_{t}}{3}$
SM_t_ = $\frac{\sum_{i=0}^{n-1}M_{t-i}}{n}$
D_t_ = $\frac{\sum_{i=0}^{n-1}|M_{t-i}-SM_{t}|}{n}$

**Table 2:** Summary statistics of indicators.

Feature	Max	Min	Mean	Standard Deviation
Diversified Financials				
SMA	6969.46	227.5	1471.201	1196.926
WMA	3672.226	119.1419	772.5263	630.0753
MOM	970.8	−1017.8	21.77033	126.5205
STCK	99.93224	0.159245	53.38083	19.18339
STCD	96.9948	14.31843	53.34332	15.28929
RSI	68.96463	27.21497	50.18898	6.471652
SIG	310.5154	−58.4724	16.64652	51.62368
LWR	99.84076	0.06776	46.61917	19.18339
ADO	0.99986	0.000682	0.504808	0.238426
CCI	270.5349	−265.544	14.68813	101.8721
Basic Metals				
SMA	322,111.5	7976.93	69,284.11	60,220.95
WMA	169,013.9	4179.439	36,381.48	31,677.51
MOM	39,393.8	−20,653.8	1030.265	4457.872
STCK	98.47765	1.028891	54.64576	16.41241
STCD	90.93235	12.94656	54.64294	13.25043
RSI	72.18141	27.34428	49.8294	6.113667
SIG	12,417.1	−4019.14	803.5174	2155.701
LWR	98.97111	1.522349	45.36526	16.43646
ADO	0.999141	0.00097	0.498722	0.234644
CCI	264.6937	−242.589	23.4683	99.14922
Non-metallic Minerals				
SMA	15,393.62	134.15	1872.483	2410.316
WMA	8081.05	69.72762	985.1065	1272.247
MOM	1726.5	−2998.3	49.21097	264.0393
STCK	100.00	0.154268	54.71477	20.2825
STCD	96.7883	13.15626	54.68918	16.37712
RSI	70.89401	24.07408	49.67247	6.449379
SIG	848.558	−127.47	37.36441	123.9744
LWR	99.84573	−2.66648	45.28523	20.2825
ADO	0.998941	0.00036	0.501229	0.238008
CCI	296.651	−253.214	20.06145	101.9735
Petroleum				
SMA	1,349,138	16,056.48	243,334.2	262,509.8
WMA	707,796.4	8580.536	127,839.1	138,101
MOM	227,794	−136,467	4352.208	26,797.25
STCK	100.00	0.253489	53.78946	22.0595
STCD	95.93565	2.539517	53.83312	17.46646
RSI	75.05218	23.26627	50.02778	6.838486
SIG	71830.91	−33132	3411.408	11,537.98
LWR	99.74651	−1.8345	46.23697	22.02162
ADO	0.999933	0.000288	0.498381	0.239229
CCI	286.7812	−284.298	14.79592	101.8417

**Table 3:** Tree-based models parameters.

Model	Parameters	Value(s)
Decision Tree	Number of Trees (ntrees)	1
Bagging	Number of Trees (ntrees)	50, 100, 150, 200, 250, 300, 350, 400, 450, 500
	Max Depth	10
Random Forest	Number of Trees (ntrees)	50, 100, 150, 200, 250, 300, 350, 400, 450, 500
	Max Depth	10
Adaboost	Number of Trees (ntrees)	50, 100, 150, 200, 250, 300, 350, 400, 450, 500
	Max Depth	10
	Learning Rate	0.1
Gradient Boosting	Number of Trees (ntrees)	50, 100, 150, 200, 250, 300, 350, 400, 450, 500
	Max Depth	10
	Learning Rate	0.1
XGBoost	Number of Trees (ntrees)	50, 100, 150, 200, 250, 300, 350, 400, 450, 500
	Max Depth	10
	Learning Rate	0.1

**Table 4:** Neural network-based models parameters.

Model	Parameters	Value(s)
Artificial neural networks (ANN)	Number of Neurons	500
	Activation Function	Relu
	Optimizer	Adam ($\beta_{1}=0.9,\;\beta_{2}=0.999$)
	Learning Rate	0.01
	Epochs	100, 200, 500, 1000
Recurrent neural network (RNN)	Number of Neurons	500
	Activation Function	tanh
	Optimizer	Adam ($\beta_{1}=0.9,\;\beta_{2}=0.999$)
	Learning Rate	0.0001
	Training Days (ndays)	1, 2, 5, 10, 20, 30
	Epochs (w.r.t. ndays)	100, 200, 300, 500, 800, 1000
Long short-term memory (LSTM)	Number of Neurons	200
	Activation Function	tanh
	Optimizer	Adam ($\beta_{1}=0.9,\;\beta_{2}=0.999$)
	Learning Rate	0.0005
	Training Days (ndays)	1, 2, 5, 10, 20, 30
	Epochs (w.r.t. ndays)	50, 50, 70, 100, 200, 300

**Table 5:** Diversified financials one day ahead.

Prediction Models	Parameters	Error Measures			
		MAPE	MAE	rRMSE	MSE
	ntrees				
Decision Tree	1	1.29	23.05	0.0235	4948.07
Bagging	400	0.92	15.80	0.0142	1403.24
Random Forest	300	0.92	15.51	0.0141	1290.91
Adaboost	250	0.91	15.09	0.0132	912.51
Gradient Boosting	300	1.02	19.19	0.0203	4312.09
**XGBoost**	**100**	**0.88**	**14.86**	**0.0120**	**804.97**
	epochs				
ANN	1000	1.01	16.07	0.0146	1107.02
	ndays				
RNN	1	1.59	14.70	0.0242	362.26
**LSTM**	**5**	**0.43**	**4.46**	**0.0065**	**48.06**

**Table 6:** Diversified financials two days ahead.

Prediction Models	Parameters	Error Measures			
		MAPE	MAE	rRMSE	MSE
	ntrees				
Decision Tree	1	1.52	25.93	0.0250	2893.88
**Bagging**	**150**	**1.10**	**18.31**	**0.0160**	**1320.55**
Random Forest	500	1.12	18.39	0.0171	1322.25
Adaboost	400	1.11	19.56	0.0164	1687.85
Gradient Boosting	300	1.14	19.51	0.0199	1781.31
XGBoost	150	1.14	19.81	0.0162	1724.65
	epochs				
ANN	1000	1.41	23.35	0.0208	2614.08
	ndays				
RNN	10	1.66	14.75	0.0243	423.14
**LSTM**	**2**	**0.54**	**5.21**	**0.0076**	**72.71**

**Table 7:** Diversified financials five days ahead.

Prediction Models	Parameters	Error Measures			
		MAPE	MAE	rRMSE	MSE
	ntrees				
Decision Tree	1	1.66	28.94	0.0298	4715.42
Bagging	150	1.45	24.00	0.0215	2146.32
Random Forest	500	1.47	24.46	0.0216	2317.71
Adaboost	400	1.39	23.91	0.0198	2494.78
**Gradient Boosting**	**350**	**1.35**	**23.05**	**0.0142**	**2002.51**
XGBoost	300	1.45	24.12	0.0202	2056.23
	epochs				
ANN	1000	2.27	39.69	0.0322	7156.56
	ndays				
RNN	10	1.77	15.21	0.0263	468.32
**LSTM**	**30**	**0.55**	**6.02**	**0.0077**	**91.66**

**Table 8:** Diversified financials 10 days ahead.

Prediction Models	Parameters	Error Measures			
		MAPE	MAE	rRMSE	MSE
	ntrees				
Decision Tree	1	2.09	34.00	0.0382	5129.32
Bagging	250	1.88	31.47	0.0283	3219.30
Random Forest	300	1.86	31.36	0.0279	3246.80
**Adaboost**	**200**	**1.58**	**25.63**	**0.0251**	**2122.81**
Gradient Boosting	500	1.74	28.00	0.0322	3356.01
XGBoost	500	1.77	31.07	0.0257	3600.53
	epochs				
ANN	1000	4.12	65.38	0.0556	18,866.04
	ndays				
RNN	5	1.91	16.98	0.0280	528.71
**LSTM**	**10**	**0.57**	**6.84**	**0.0087**	**131.16**

**Table 9:** Diversified financials 15 days ahead.

Prediction Models	Parameters	Error Measures			
		MAPE	MAE	rRMSE	MSE
	ntrees				
Decision Tree	1	2.28	41.29	0.0451	11,051.93
Bagging	100	2.24	37.61	0.0349	4997.20
Random Forest	50	2.24	37.28	0.0349	4755.32
**Adaboost**	**300**	**1.83**	**28.83**	**0.0301**	**3146.31**
Gradient Boosting	200	1.97	35.95	0.0390	8759.44
XGBoost	500	2.03	35.37	0.0305	5534.65
	epochs				
ANN	1000	5.05	85.46	0.0696	29,483.87
	ndays				
RNN	10	1.95	19.09	0.0307	644.50
**LSTM**	**20**	**0.61**	**7.06**	**0.0112**	**150.86**

**Table 10:** Diversified financials 20 days ahead.

Prediction Models	Parameters	Error Measures			
		MAPE	MAE	rRMSE	MSE
	ntrees				
Decision Tree	1	2.80	49.12	0.0571	14,227.06
Bagging	100	2.56	42.43	0.0388	5916.19
Random Forest	450	2.57	42.66	0.0393	6008.33
**Adaboost**	**450**	**2.01**	**33.25**	**0.0309**	**4340.09**
Gradient Boosting	350	2.17	39.10	0.0385	8573.37
XGBoost	500	2.30	39.30	0.0358	6406.16
	epochs				
ANN	1000	5.66	126.69	0.0790	42,701.88
	ndays				
RNN	20	1.96	19.47	0.0314	668.82
**LSTM**	**10**	**0.75**	**7.25**	**0.0113**	**170.14**

**Table 11:** Diversified financials 30 days ahead.

Prediction Models	Parameters	Error Measures			
		MAPE	MAE	rRMSE	MSE
	ntrees				
Decision Tree	1	2.83	48.39	0.0587	12,924.43
Bagging	350	3.21	54.37	0.0467	8803.66
Random Forest	50	3.18	54.06	0.0465	8799.45
**Adaboost**	**350**	**2.33**	**37.63**	**0.0374**	**5369.06**
Gradient Boosting	500	2.54	43.59	0.0485	9354.03
XGBoost	400	2.48	42.85	0.0378	6306.78
	epochs				
ANN	1000	7.48	126.69	0.0994	54,940.25
	ndays				
RNN	20	2.11	20.20	0.0322	1355.35
**LSTM**	**10**	**0.77**	**10.03**	**0.0121**	**376.82**

**Table 12:** Average performance for diversified financials.

Prediction Models	Error Measures			
	MAPE	MAE	rRMSE	MSE
Decision Tree	2.07	35.82	0.0396	7984.30
Bagging	1.91	32.00	0.0288	3973.92
Random Forest	1.91	31.96	0.0288	3962.97
**Adaboost**	**1.59**	**26.27**	**0.0248**	**2867.63**
Gradient Boosting	1.70	29.91	0.0318	5662.68
XGBoost	1.72	29.63	0.0255	3776.28
ANN	3.86	69.05	0.0530	22,409.96
RNN	1.85	17.20	0.0281	635.85
**LSTM**	**0.60**	**6.70**	**0.0093**	**148.77**

**Table 13:** Average performance for Petroleum.

Prediction Models	Error Measures			
	MAPE	MAE	rRMSE	MSE
Decision Tree	2.70	7613.54	0.0528	502,831,775.59
Bagging	2.62	6640.41	0.0397	212,982,692.85
Random Forest	2.62	6649.18	0.0400	212,239,589.62
**Adaboost**	**2.22**	**5279.15**	**0.0362**	**163,264,613.31**
Gradient Boosting	2.26	6402.08	0.0403	305,274,334.62
XGBoost	2.33	5947.22	0.0363	175,385,973.35
ANN	5.52	14,045.78	0.0753	1,123,371,989.92
RNN	3.40	4097.20	0.0596	57,606,535.91
**LSTM**	**1.18**	**1653.79**	**0.0198**	**8,175,371.29**

**Table 14:** Average performance for Non-metallic minerals.

Prediction Models	Error Measures			
	MAPE	MAE	rRMSE	MSE
Decision Tree	2.18	52.75	0.0456	22,287.11
Bagging	2.12	47.88	0.0331	13,333.59
Random Forest	2.12	47.89	0.0331	13,045.77
**Adaboost**	1.84	**41.31**	**0.0305**	11,798.23
**Gradient Boosting**	**1.78**	43.26	0.0339	15,155.18
**XGBoost**	1.86	42.15	0.0312	**10,815.16**
ANN	4.67	100.28	0.0662	98,705.75
RNN	5.23	44.18	0.0875	9227.55
**LSTM**	**1.52**	**16.94**	**0.0228**	**1289.28**

**Table 15:** Average performance for Metals.

Prediction Models	Error Measures			
	MAPE	MAE	rRMSE	MSE
Decision Tree	1.41	1159.46	0.0274	11,082,872.18
Bagging	1.36	1046.64	0.0207	5,314,782.99
Random Forest	1.36	1043.30	0.0207	5,192,173.88
**Adaboost**	1.18	**862.73**	**0.0191**	**3,361,111.64**
**Gradient Boosting**	**1.16**	960.52	0.0212	7,029,319.85
**XGBoost**	1.21	963.42	**0.0191**	4,619,506.50
ANN	3.17	2441.71	0.0420	31,250,640.68
RNN	1.48	663.45	0.0238	1,434,974.44
**LSTM**	**0.54**	**272.95**	**0.0077**	**225,333.35**

**Table 16:** Average runtime per sample for all models.

Tree-Based						
Models	Decision Tree	Bagging	Random Forest	Adaboost	Gradient Boosting	XGBoost
Average runtime per sample (ms)	0.009	1.399	1.316	1.308	1.483	2.373
ANNs-based						
Models	ANN		RNN		LSTM	
Average runtime per sample (ms)	20.088		20.630		80.902	
